# Pre-Stimulus Activity of Left and Right TPJ in Linguistic Predictive Processing: A MEG Study

**DOI:** 10.3390/brainsci14101014

**Published:** 2024-10-10

**Authors:** Sara Lago, Sara Zago, Valentina Bambini, Giorgio Arcara

**Affiliations:** 1 IRCCS San Camillo Hospital, 30126 Venice, Italy; sara.lago@hsancamillo.it (S.L.); sara.zago@hsancamillo.it (S.Z.); 2 Padova Neuroscience Center, University of Padua, 35129 Padua, Italy; 3 Laboratory of Neurolinguistics and Experimental Pragmatics (NEPLab), Department of Humanities and Life Sciences, University School for Advanced Studies IUSS, 27100 Pavia, Italy; valentina.bambini@iusspavia.it

**Keywords:** predictive processing, language, MEG, magnetoencephalography, temporo-parietal junctions, TPJs, pre-stimulus alpha, metaphor, figurative language, neuropragmatics

## Abstract

Background. The left and right temporoparietal junctions (TPJs) are two brain areas involved in several brain networks, largely studied for their diverse roles, from attentional orientation to theory of mind and, recently, predictive processing. In predictive processing, one crucial concept is prior precision, that is, the reliability of the predictions of incoming stimuli. This has been linked with modulations of alpha power as measured with electrophysiological techniques, but TPJs have seldom been studied in this framework. Methods. The present article investigates, using magnetoencephalography, whether spontaneous oscillations in pre-stimulus alpha power in the left and right TPJs can modulate brain responses during a linguistic task that requires predictive processing in literal and non-literal sentences. Results. Overall, results show that pre-stimulus alpha power in the rTPJ was associated with post-stimulus responses only in the left superior temporal gyrus, while lTPJ pre-stimulus alpha power was associated with post-stimulus activity in Broca’s area, left middle temporal gyrus, and left superior temporal gyrus. Conclusions. We conclude that both the right and left TPJs have a role in linguistic prediction, involving a network of core language regions, with differences across brain areas and linguistic conditions that can be parsimoniously explained in the context of predictive processing.

## 1. Introduction

### 1.1. The Many Facets of the TPJs

The term temporoparietal junctions (TPJs) refers to bilateral portions of the cortex, approximately including the inferior parietal lobules (supramarginal and angular gyri) and extending into the posterior sections of the superior temporal gyri [1,2]. In spite of their relatively small size, the TPJs were found to play a role in a variety of cognitive processes; for instance, attention [3,4], language (especially left TPJ [5]), body ownership and sense of agency [6,7], and episodic memory retrieval [8,9]. Bilateral TPJs are also part of the default mode network (DMN), which is active during social cognition tasks [10,11].

The two TPJs (left and right) show some specificity in their functional roles. For example, attentional tasks seem to activate more specifically the right TPJ (rTPJ), especially in response to unexpected but task-relevant stimuli [3,4]. This led to the hypothesis that rTPJ could play a key role in the reorienting of attention [12,13]. This hypothesis, known as the circuit-breaking theory, states that the dorsal attention network (DAN) maintains the visuospatial information relevant to the current task-defined goals, while the ventral attention network (VAN), including the rTPJ, allows for the switching of attention to relevant but currently unattended stimuli; the rTPJ would therefore be responsible for interrupting the activity of the DAN, resulting in the reorientation of attention to a new salient stimulus [12,13].

Conversely, the left TPJ (lTPJ) appears to be more involved in language processing: it is activated in response to lexical violations, non-words, and words that are semantically unrelated to the previous context [14,15,16], but it is also in charge of integrating the individual’s general world knowledge with the local discourse information [17,18] and in pre-activating linguistic information [5].

### 1.2. TPJs as Predictive Hubs: Literature’s Findings

The involvement of the bilateral TPJs in such heterogeneous contexts has led to some domain-general conceptualizations of the role of these areas, in opposition to the domain-specific approaches outlined so far, which focus on each cognitive ability separately. Domain-general (or domain-independent) approaches strive to streamline neurobiological mechanisms that are common to multiple areas of interest, such as perception, action, and cognition [19,20].

For example, the nexus model [21] aims at reconciling the roles of the TPJs in social cognition and attention within a comprehensive theory. According to this model, the TPJs serve as a central hub, or “nexus”, where lower-level functions such as attentional reorientation intersect with higher-order social-cognitive functions. The central concept of the nexus model is that the TPJs integrate information from various cognitive domains to create a social framework that supports decision-making.

Another overarching theory built upon the concept that the TPJs are integrative hubs was proposed by Geng and Vossel [22]. Their theory is based on the functional interpretation of the P300, an event-related potential (ERP) component that arises from various neural sources, and in particular of the P3b, a subcomponent of the P300. This subcomponent was linked to the TPJs and is considered a neural marker of contextual updating [23,24], i.e., the adjustment of the individual’s representation of the environmental context. According to this view, known as the contextual updating hypothesis, the TPJs play a role in updating internal models (representations) of the environment, which in turn generate appropriate expectations and behaviors [22]. Similarly to the nexus model, the contextual updating hypothesis provides a framework for understanding both basic functions, such as reorienting attention, and complex social functions, such as Theory of Mind (ToM), pointing to contextual updating as a shared computational process [2].

In sum, these more general theories state that the TPJs, in particular the rTPJ, would specifically act as a hub for integrating information from multiple domains and thus updating the internal models of the world.

#### 1.2.1. Predictive Processing

Predictive processing theories fall into the category of domain-general conceptualizations, as they aim to provide comprehensive frameworks for understanding the neural processes involved in perception, cognition, and action [25,26]. These theories vary greatly, but they tend to share some general features, for example, the concept of internal models, i.e., representations of the environmental stimuli and/or the individual’s inner states [25], and they agree that the brain employs internal models to infer the underlying causes of sensory (or internal) inputs with the ultimate goal of anticipating and adapting to changes in the environment or in the individual’s inner states [25,27]. Internal models spontaneously analyze sensory inputs to extract relevant causal regularities, allowing the cognitive system to generate predictions (also known as “priors”) about upcoming events and detect inconsistencies between these predictions and the actual sensory information. When a prediction fails to match the sensory input, a prediction error occurs. Prediction errors are then used to update and improve subsequent predictions, which are in turn tested against the actual input (henceforth called “prediction testing”), in a continuous cycle that aims at minimizing prediction error and refining hypotheses about the cause of the sensory stimuli [28]. Such predictive computations are believed to be carried out in a distributed way across the brain and at different hierarchical levels: a recent meta-analysis proposed that a set of brain areas, comprising also the rTPJ, form a diffuse network involved in higher-level prediction generation and testing which supports both perception and action processes in a domain-general way [29].

Besides the putative prediction network, the rTPJ is part of both the VAN and the DMN [10,12], a co-occurrence that further speaks in favor of a domain-general conceptualization of this area as a hub where multiple cognitive processes and information types converge and are integrated. In this vein, there is also evidence from neurological patients that damage to the rTPJ or disconnection of this area from other brain regions has a disruptive impact on several aspects of perception, cognition, and motion, causing cognitive impairments that could be interpreted as aberrant predictive processing [2]. Interestingly, reduced functional connectivity between the rTPJ (but also the left) and other brain areas was shown to impact specifically pragmatics and figurative language processing in neurodegenerative patients [30].

#### 1.2.2. Linguistic Predictive Processing in Literal and Metaphorical Expressions

Predictive processing in the language domain has been extensively and increasingly studied in recent years [31,32]. Adapting the predictive processing general principles to the language domain, we can infer that during linguistic prediction, our internal models test the possible sentence meaning against our prior knowledge of the world and the actual sensory input until we come up with a plausible interpretation. This requires integrating global world knowledge and local sentence information, and unsurprisingly, beyond more classical linguistic areas, the lTPJ seems to be particularly implicated in this process [1,5,17,18].

Notably, a special case of linguistic prediction can occur in figurative uses of language [33]. Figurative language includes various forms, e.g., metaphors, idioms, and irony, all expressions where the intended meaning goes beyond the literal content of the single sentence components [34,35]. For instance, metaphors offer concise and evocative descriptions by creatively connecting two different concepts that share some features (e.g., “that lawyer is a shark”, meaning that the lawyer is aggressive). Differently from literal expressions, figurative ones capitalize on both literal and non-literal meanings [36], and the principles of predictive processing can help us understand how we make sense of figurative language.

In the context of the Rational Speech Act framework, Goodman and Frank [37] argued that speakers produce sentences that are both helpful and parsimonious, relative to some particular topic or goal. Comprehenders then understand these sentences by inferring what the speaker must have meant, given what she said. This means that comprehenders infer the underlying cause (what the speaker intends to communicate) based on the sensory input (what the speaker said). Expanding on this observation, we can hypothesize that in the case of metaphors, to correctly interpret the intended meaning of the sentence, i.e., to infer the figurative meaning beyond the literal one, we have to rely on some expectations coming from our prior knowledge, as proposed in computational models of figurative meaning [38,39,40]. Consider the sentence “that lawyer is a shark”. While reading it, we generate a hypothesis about the general meaning of the sentence based on our prior knowledge about lawyers. We are, in other words, uncertain about the plausible implied meaning of the sentence [31], but we have some priors, coming from our general knowledge, that can help us in the interpretation. Thanks to these priors, we can identify the relevant features of the concept of “shark” in the context of the expression, such as, for example, its ferocity or aggressiveness. In addition, during predictive linguistic computations, the degree of uncertainty (i.e., precision) associated with our prediction about the meaning can vary, but usually, our priors guide us through the inference with a fair degree of precision and are involved in updating our internal models of the sentence. The concept of precision mathematically represents the reliability of a prediction and is defined as the prediction’s inverse variance [41]. In essence, the higher the precision of a stimulus or expectation, the more the individual relies on it; therefore, highly precise expectations reduce the uncertainty associated with the underlying cause of upcoming stimuli (in the case of figurative language comprehension, about the most plausible meaning of a metaphor). Interestingly, there is evidence showing the involvement of the lTPJ in figurative language comprehension [42] but there is also evidence that this type of task activates the TPJs bilaterally [43,44].

#### 1.2.3. Are TPJs Really Involved in Prediction?

Recent evidence suggests that the TPJs are involved in predictive computations across several cognitive domains [1,2,29]. However, these studies, together with those reviewed so far, have mostly detected TPJ activation after predictions were disconfirmed, only assuming that the detected mismatch was the consequence of a violated prediction, but not directly investigating whether a prediction is actually taking place (a limitation shared with many studies on linguistic prediction [45,46]). Based on this evidence, we can argue that the TPJs are involved in prediction violation, rather than in prediction per se; moreover, the rTPJ has been studied in the context of predictive processing more frequently than its left-hemisphere counterpart, which could also have a role in prediction. Hence, some questions arise: are both TPJs involved in prediction? And do the left and right TPJ have different roles in predictive computations? Answering these questions implies investigating the TPJs’ activity during cognitive tasks, not only after target stimuli but also before them. We, therefore, direct our interest toward the TPJs’ state during the pre-stimulus interval, which could be representative of prediction processes, and, as such, might modulate the neural responses to subsequent stimuli.

#### 1.2.4. The Pre-Stimulus Interval: Alpha, Precision, and Attention

Some evidence from electroencephalographic (EEG) studies shows that different levels of pre-stimulus alpha power (8–13 Hz) might modulate post-stimulus ERPs: in simple perceptual paradigms, lower levels of pre-stimulus alpha power have been linked to larger ERP amplitudes [47,48,49,50,51], while higher pre-stimulus alpha power has been associated with ERP amplitude suppression, longer latencies, and augmentation of late ERPs (occurring after 400 ms [52,53,54]). This evidence does not give us any insight into the role of the TPJs, but it suggests that spontaneous fluctuations of pre-stimulus alpha power can influence stimulus processing and that they can be interpreted as possible correlates of anticipation or predictive processing [55,56,57,58].

Alpha power has been traditionally considered indicative of anticipatory attentional suppression [59], controlling the redirection of information flow to brain areas relevant to the task at hand, while inhibiting irrelevant regions [60]. Under this perspective, increases in alpha power before a target stimulus suggest a state of cortical inhibition, whereas decreases in alpha power indicate cortical activation, facilitating the subsequent detection and processing of task-relevant information [61]. In sum, during a state of anticipatory attention or cortical activation, stimulus processing should be facilitated; in this context, an unexpected stimulus would elicit an enhanced ERP.

On the other hand, a predictive conceptualization of alpha oscillations proposes that they are linked to the concept of precision (see Section 1.3 [62,63,64]). Notably, in the context of predictive processing theories, attention is regarded as an emergent property of the precision optimization mechanism, where directing attention to a stimulus involves representing and enhancing the precision of sensory information (including prediction error) throughout the inferential process [65]. As mentioned above, highly precise expectations reduce the uncertainty associated with the underlying cause of upcoming stimuli (in the previously described case of metaphor comprehension, about the most plausible meaning); as a consequence, the relative internal representations will be enhanced and so will the neural responses associated with them. In this way, cognitive resources will be biased towards more precise predictions [66], and the magnitude of expectations’ effects on neural activity depends on the precision of predictions and prediction error signals [67]. Within this theoretical framework, alpha oscillations are considered correlates of the prior’s precision and capable of modulating post-stimulus neural responses, and there appears to be a correlation between highly precise (reliable) predictions and reduced pre-stimulus alpha power [47,63]. Conversely, elevated levels of pre-stimulus alpha power have been linked to dampened stimulus-evoked responses [47]. It is however important to note that variations in pre-stimulus alpha power can also arise spontaneously, without any experimental manipulations [68,69]; additionally, within a specific task, the features of the stimuli do not exhibit a linear relationship with the precision of their internal representations, as predictive models include random fluctuations of precision (state-dependent error variance) and assume that this precision is not constant at any level of hierarchical inference [65]. As a result, priors’ precision levels may fluctuate spontaneously during the task, similar to attention and alpha power [45].

This view of alpha oscillations aligns also with traditional “inhibitory” theories of attention, stating that anticipatory allocation of cognitive resources is reflected in lower levels of alpha power, facilitates stimulus processing, and results in enhanced ERPs. Nevertheless, predictive processing differs from traditional accounts as it proposes a continuous interplay between attention and expectation that is aimed at optimizing precision and minimizing prediction error [67]. Interestingly enough, Siman-Tov et al. [29] substantiate the idea that prediction and attention are interdependent processes: they showed that their putative prediction network and the VAN, subserving attentional reorienting, overlap in the rTPJ, and some of the conditions activating the VAN also involve violation of predictions [12,70]. To the best of our knowledge, no previous studies have directly investigated the influence of spontaneous (i.e., in the absence of manipulation) pre-stimulus TPJ alpha activity on post-stimulus processing, a topic that might shed light on the neurophysiological correlates of predictive dynamics unfolding in the TPJs.

#### 1.2.5. Pre-Stimulus Alpha in Language

Prediction violation is an important feature when studying predictive processing, especially in the domain of language, in which expectations are mainly studied using EEG paradigms where the final target word is incongruent with the previous sentence contexts. Typically, incongruent (and therefore unpredictable) words elicit characteristic N400s and P600s ERPs, respectively associated with linguistic prediction error and its resolution through reanalysis, repair, or reinterpretation of the input to make sense of the incongruency [32]. Similarly to what happens for the TPJs, whose role in prediction has been inferred primarily based on their involvement after disconfirmation of expectations, the main drawback of linguistic violation paradigms is that they focus on the neural dynamics occurring after predictions have been either confirmed or disconfirmed; as a result, these experiments assume that prediction is occurring before the target, but they do not directly address it [46]. To overcome this limitation, some studies on linguistic processing have considered both the pre- and post-stimulus interval dynamics, in different ways. What emerges is that more reliable, stronger linguistic expectations (induced, for example, by a more constraining context) were associated with a reduced pre-stimulus alpha power and more negative N400s [71,72,73,74]. In addition, in a semantic congruence task where image cues induced highly precise expectations about target words that could be congruent or incongruent, spontaneous fluctuations of pre-stimulus alpha power modulated the amplitude of the N400 and P600 components: the lower the pre-stimulus power, the greater the ERP amplitudes [45]. Such influence of fluctuations in pre-stimulus alpha power on the subsequent neural responses supports the hypothesis that alpha oscillations are associated with the prediction’s precision level, which modulates how the upcoming stimuli will be processed. Alpha oscillations can therefore be interpreted as variations in the individual’s ability to predict the upcoming target word with a high level of precision [47].

This mechanism could be consistent with the predictive processing account of metaphor interpretation as outlined in Section 1.2.2: linguistic predictions toward the most plausible interpretation, guided by prior world knowledge, might be associated with varying levels of precision and, therefore, with varying levels of pre-stimulus alpha power. Where and how this mechanism is carried out in the brain remains an open question. For example, linguistic prediction could only be carried out within the language network; or maybe these computations might also involve other domain-general predictive hubs, which are functionally connected with the language network and, probably, are also active in other cognitive operations [75].

As reviewed so far, there are reasons to hypothesize that, even if they are not strictly part of the language network, the TPJs (or at least the left one) might come into play during linguistic prediction. If, as some studies report [1,29], the TPJs are involved in domain-general prediction and they are functionally connected with task-specific areas [2], we can hypothesize that pre-stimulus alpha power recorded in the TPJs (associated with the prior’s precision) could modulate the neural responses to language understanding, and in particular metaphor comprehension.

### 1.3. The Present Study

To sum up, in predictive frameworks, fluctuations in precision have been linked with spontaneous pre-stimulus alpha oscillations, which can modulate the post-stimulus EEG responses; in addition, the prior’s precision guides resource allocation, probably with the involvement of the rTPJ (the overlapping point between the VAN, a domain-specific network engaged in attentional processes, and a putative domain-general prediction network). Such evidence points to the importance of considering, when studying predictive dynamics, both fluctuations in precision variations and the potential functional connections between rTPJ and other areas. This last consideration is also valid for the lTPJ, which has been somewhat overlooked in the investigations about predictive processing. In addition, the TPJs have been rarely studied in the context of linguistic prediction [5]. This is striking, as there is evidence that the rTPJ may be part of a predictive network and that the lTPJ might be involved in (linguistic) prediction by integrating local, task-related linguistic information with our existing world knowledge, with the aim of formulating the priors guiding language comprehension [17,18]. Such predictive mechanisms in language might become especially relevant in figurative expressions, given the intrinsic gap between the literal and the intended meaning and the availability of multiple interpretations [33,39].

For these reasons, the goal of the present study was to investigate the role of the bilateral TPJs in linguistic prediction during a sentence comprehension task with literal and figurative items. More specifically, we wanted to answer the following questions:

(a) are both TPJs involved in linguistic prediction?
(b) can spontaneous fluctuations of pre-stimulus alpha (associated with predictions’ precision) recorded in the TPJs modulate the subsequent brain responses to target stimuli?
(c) is this modulation local, i.e., limited to the TPJs, or can pre-stimulus TPJ activity influence post-stimulus activations in other task-related (in this case linguistic) areas?
(d) do the left and right TPJ exert different kinds of influence on post-stimulus responses recorded from linguistic ROIs?

As stated in Section 1.2.3 and Section 1.2.5, the influence of the pre-stimulus dynamics on the post-stimulus neural responses has been seldom tested in the literature on linguistic prediction, but it might be crucial for elucidating the temporal aspects of predictive dynamics. The existing literature lacks information about the spatial aspects of this interplay, i.e., whether neural responses recorded from the language network are sensitive to pre-stimulus alpha fluctuations recorded in domain-general predictive hubs such as the TPJs.

Answering these questions might fill the gap in the existing literature about the temporal and spatial dynamics of linguistic prediction, thereby significantly advancing our knowledge in this field; however, these questions also pose some methodological challenges, relative to both data acquisition and analysis, that need to be addressed carefully. The majority of studies reviewed so far are functional magnetic resonance imaging (fMRI) studies (or meta-analyses and systematic literature reviews based on fMRI studies) with a very good spatial resolution but a limited temporal resolution, whereas the investigation of pre-stimulus TPJ dynamics requires adequate resolution in both the temporal and spatial domains. Magnetoencephalography (MEG) meets these requirements, since it allows a reliable source reconstruction of the signal together with an excellent temporal resolution, enabling the investigation of the influence of the pre-stimulus oscillations on the post-stimulus signal both within the TPJs and in connection with other task-specific, language-related areas. Concerning the areas that may be involved in the predictive processing of metaphoric sentences together with the TPJs, we chose to consider three core regions of interest (ROIs) within the language network, namely, Broca’s area and the superior and middle temporal gyri (lSTG and lMTG [76,77]).

In addition to a suitable data acquisition technique, the right statistical method is needed to achieve our research goals. For this reason, pre-stimulus alpha power and post-stimulus neural response time series extracted from each ROI during the experimental task were analyzed using Generalized Additive Mixed Models (GAMs), a technique particularly apt for considering the temporal unfolding of the neural signal [45,78]. In particular, GAMs allow to model complex non-linear relationships between predictors and the dependent variable by estimating the non-linearity in a bottom-up fashion and allowing to include both fixed and random effects [79].

In sum, the present study aimed to investigate whether pre-stimulus activity in the left and right TPJs can influence the post-stimulus neural responses recorded from core regions of the linguistic network. If confirmed, these effects could be interpreted as functional connections between the TPJs, acting as predictive hubs, and other language-related regions [1,2,29].

## 2. Materials and Methods

### 2.1. Participants

A sample of 28 healthy participants (17F; 11M) took part in the study.

This sample size is usually enough to grant adequate power in neurophysiological studies and especially MEG ones [80,81,82].

All participants had no neurological or psychiatric diseases that could affect cognitive performance and had no history of developmental dyslexia or dyscalculia. The mean age of participants was 28.14 years (SD = 5.46, range = 21–45) and their mean education was 17.25 years (SD = 2.10, range = 13–21). All participants were right-handed.

This study was approved by the local ethics committee (Comitato Etico per la Sperimentazione Clinica della provincia di Venezia e IRCCS San Camillo) and conducted following the guidelines of the Declaration of Helsinki.

### 2.2. Procedures

Participants were prepared before entering the magnetically shielded room. Three head coils were placed to monitor head position during MEG recording, and eight external electrodes were used to record vertical and horizontal eye movements and EKG (all with bipolar montage). Coil positions and head shape were digitized before recordings using the Polhemus Fastrak system.

Continuous MEG signal was acquired using a whole-head 275-channel axial gradiometer system (MISL, Vancouver, BC, Canada). Data were collected at a sampling rate of 1200 Hz, using a hardware anti-aliasing low-pass filter set at 300 Hz. Prior to the experimental session, participants completed a five-minute eyes-open resting state session, followed by the experimental task, which consisted of four runs, each lasting approximately five minutes, intermixed by breaks to allow participants to rest and adjust their positions as needed. The entire experiment lasted around 45 min. Throughout all recordings, head movements were consistently kept below a 5 mm threshold along any axis.

Additionally, all participants underwent an MRI scan for source localization purposes, using a Philips Achieva 1.5 T (Philips Medical Systems, Best, The Netherlands) with a T1-3D sequence. If a participant had already completed this scan at the institution and the data were accessible, we retrieved the existing scan. Otherwise, a new MRI scan was conducted.

### 2.3. Stimuli and Task

Participants were informed that they would be performing a language task involving the reading of short, word-by-word sentences (without any specification on the presence of figurative items). At the end of each sentence, they were presented with an adjective-matching task, being requested to select one out of two words two based on a match with the previous sentence.

The experimental items comprised 164 sentences structured as “that X is a Y”, partly retrieved from [83]. Of these, 82 sentences involved X and Y with a literal relationship (e.g., “that fish is a shark”), while the other 82 featured X and Y in a metaphorical relationship (e.g., “that lawyer is a shark”), in a paired design with the same target words across conditions (e.g., “shark”). The two conditions were equivalent for the main psycholinguistic variables (i.e., familiarity, cloze probability, and entropy), except for the semantic similarity between Xs and Ys (for details, see [84]). Some examples of stimuli can be found in Table 1.

**Figure 1:**
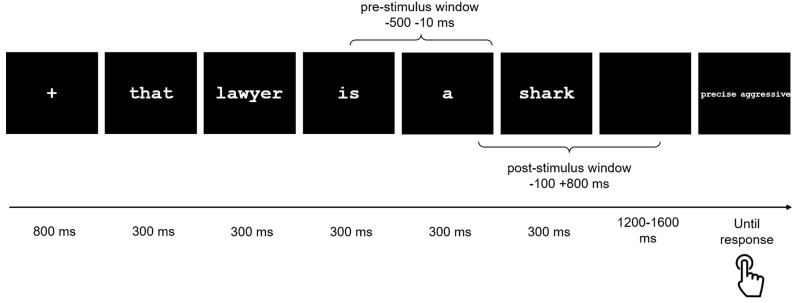
Example of a metaphor trial, with representations of the pre- and post-stimulus intervals.

Each trial proceeded as follows: a fixation cross (+) appeared for 1500 ms, followed by the presentation of a five-word sentence, with each word displayed individually on a separate screen for 300 ms, preceded by a blank screen lasting 200 ms (e.g., “That/lawyer/is/a/shark”). After the complete sentence, a blank screen was shown for 1200 ms to 1600 ms, followed by two words, one on the left and one on the right of the screen (e.g., “precise”, “aggressive”). An example of a metaphor trial is depicted in Figure 1. Participants were instructed to respond as quickly and accurately as possible by pressing one of two buttons with their index or middle finger, choosing the word more closely related to the sentence meaning. The hand used for the task was counterbalanced among participants, and the buttons corresponded to the spatial location of the words (left or right). The experiment was programmed using Psychopy [85] (version 1.82) on a PC.

Stimuli were divided into two lists (A and B), each containing an equal number of literal sentences and metaphors. This ensured that each target word appeared only once per list, either in a literal or metaphorical condition. Each participant was administered one of the lists, assigned in a counterbalanced manner. To disguise the focus on metaphors, the final stimuli list included 64 filler sentences, which were additional literal sentences similar to the target stimuli. These fillers were excluded from statistical analysis.

The MEG and behavioral data with a focus on post-stimulus responses and the relationship with individual characteristics were analyzed in a separate study which did not investigate the role of pre-stimulus alpha power on post-stimulus response [84].

### 2.4. MEG Data Analyses

**Figure 2:**
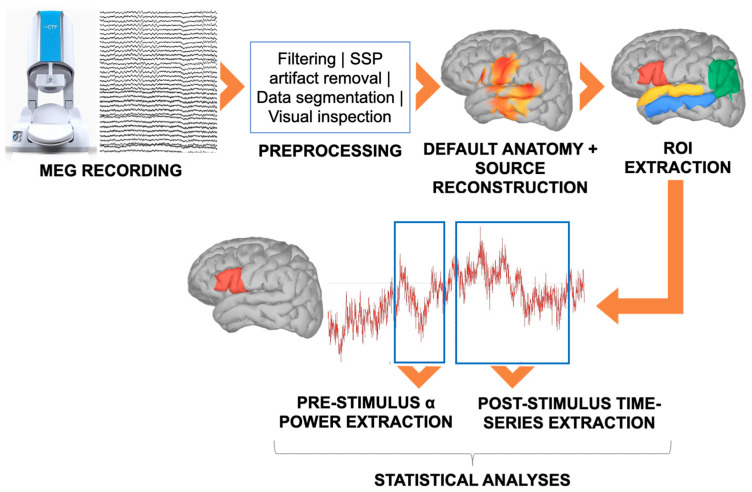
Data collection and analysis steps. The figure shows the main steps in data collection and analysis. After collection, data were cleaned and epoched. MEG signal was source reconstructed and, as a final step before statistical analyses, single-trial ROI pre-stimulus alpha power and post-stimulus activation time series were extracted.

The preprocessing of MEG data (Figure 2) was performed using Brainstorm [86] (November 2018 version) within MATLAB 2016b (Mathworks, Inc., Natick, MA, USA). Brainstorm is freely available for download under the GNU General Public License at http://neuroimage.usc.edu/brainstorm. Initially, we applied the 3rd gradient noise cancellation on the continuous recordings. Then, we resampled the data to 600 Hz and processed them with a notch filter to remove frequencies at 50 Hz and its harmonics (100, 150, 200, and 250 Hz) along with a high-pass filter set at 0.1 Hz. Cardiac and eye movement artifacts were identified and eliminated utilizing the Signal-Space Projection (SSP) algorithm. Finally, digital triggers were adjusted offline to align precisely with the actual visual stimulus presentation, thereby enhancing the accuracy of the trigger timing.

At this stage, we extracted epochs that were time-locked to the onset of the target word, with epoch durations spanning from −1500 ms to 1500 ms post-stimulus. Excessive artifacts were visually inspected and discarded during this phase. On average, each participant retained 36.61 epochs (SD = 2.2) for the Literal condition (range = 29–40) and 36.79 epochs (SD = 2.74) for the Metaphorical condition (range = 30–40), with no significant difference between the two conditions (t(51.59) = −0.27, *p* = 0.79).

The MEG forward model was created using the Boundary Element Method (BEM) based on Brainstorm’s default anatomy. Source reconstruction was conducted on the cortical surface using the weighted Minimum Norm Estimation (wMNE) algorithm, applying Brainstorm’s default settings: fixed source orientation, dipoles constrained to be normal to the cortex, depth weighting with Order [0, 1] set at 0.5 and maximal amount set at 10, noise covariance regularization at 0.1, and the regularization parameter 1/λ determined by setting the Signal-To-Noise Ratio to 3. The noise covariance was calculated from a 3-minute empty room recording made at the end of each participant’s session.

Single-trial regions of interest (ROIs) activation time series were reconstructed into 5 cortical ROIs selected from the Destrieux atlas [87] and dimension-reduced by averaging all signals within each ROI. As language-related areas, we chose the left superior and middle temporal gyri (lSTG and lMTG) and Broca’s area. The lSTG and lMTG were selected as depicted in the atlas, while Broca’s area was obtained by merging the pars opercularis and triangularis [88]. Regarding the TPJs, we took into consideration those portions of this broad region that, according to Doricchi and colleagues [1], are maximally involved in linguistic computations, since the task of interest was a linguistic one. Bilateral TPJs were thus obtained by merging two adjacent regions, namely the posterior part of the lSTG and the angular gyrus (AG [1]), which correspond to cytoarchitectonic areas PGp, PGa (angular gyrus), PF, PFcm, and PFm (posterior lSTG). MNI centroid coordinates for each ROI are reported in Table 2.

We standardized the single-trial, source-reconstructed data through Z-transformation according to the mean value of the 100 msec period preceding the target word, then downsampled to 100 Hz to reduce computational load and filtered with a 50 Hz low-pass filter to prevent aliasing. Subsequently, the data were exported into R [89] (version 3.4.4) using the erpR package [90] (version 0.2.0) for further statistical analyses. Custom code was developed for data handling and plotting.

The preprocessing for the resting-state data was identical to that of the task data. Following the artifact removal through SSP, the continuous recordings were divided into 3-s epochs. Trial rejection was then conducted based on visual inspection, similar to the process applied in the task recordings.

### 2.5. Single-Trial Time–Frequency Analysis

We conducted time–frequency (TF) analyses on source-reconstructed data at the single-trial level. A Morlet wavelet was constructed with a central frequency of 1 Hz and a time resolution of 3 s (full-width half-maximum, FWHM). The wavelet spanned from 1 to 45 Hz, with 1 Hz linear frequency steps. We selected this frequency range to allow for additional analyses beyond the scope of this paper, which will not be presented here. From the single-trial time–frequency decomposition, we extracted the average magnitude in the alpha band (8–13 Hz) and exported the single-trial alpha magnitudes to R for statistical analyses. For the resting-state recordings (previously segmented into 3-s epochs for trial rejection purposes), we followed a similar procedure, with the exception that time-resolved alpha-band magnitudes in the single epochs were first averaged in time to obtain a Morlet-based frequency spectrum. Subsequently, these values were averaged across epochs and exported. We opted to use the average resting alpha power as a baseline, as opposed to other inter-trial intervals, because participants were instructed to blink during these intervals to reduce contamination during the experimental trials. As a result, power estimates from this time window would have been affected by such artifacts.

We obtained pre-stimulus power values for each trial by averaging the TF values across the time window between −500 and −10 ms before the target word. We chose this interval length, even if not event-free, to obtain a reliable estimation of alpha power. It is important to note that there is evidence of specific anticipatory processes happening already at the level of the article during sentence reading [91], which might modulate pre-stimulus alpha power. Formally, the possible influence of the article included in this interval (“a/an”) on alpha power is not controlled, but we overcame such limitation by means of control analyses (reported in the Additional Analysis in the OSF repository: https://osf.io/y2efz/?view_only=1be400a47cd44f32814d545978cfa09d) demonstrating that none of the experimental items are systematically associated with a particularly lower (or higher) value of alpha. We can therefore rest assured that the uncontrolled properties of the stimuli did not significantly influence the level of pre-stimulus alpha power. Moreover, the words included in this interval (“is a/an”) are not content words and they are consistent throughout the sentence stimuli, so their effect on sentence comprehension should be constant across all sentences. In addition, the target word is always in agreement with the article, so sentences do not generate syntactic or semantic violations. By ending the time window 10 ms before the target, we can prevent undesired effects of temporal smearing caused by time–frequency analyses, like the temporal leakage of stimulus-related oscillatory activity into the pre-stimulus time window, particularly when the stimulus occurs at time 0 or shortly thereafter [91].

As the absolute single-trial power value might be influenced by irrelevant factors (e.g., individual participant characteristics, signal quality, etc.), we normalized the pre-stimulus power with reference to the resting-state, used as a baseline. This procedure involved dividing the power during the task by the average power during the baseline period (i.e., the resting-state recording). The resulting unit of this normalization is a ratio [92]. Values smaller than 1 indicate that during each pre-stimulus period, a given subject had lower alpha power than during the resting state; values larger than 1 indicate that the subject’s pre-stimulus power was higher than the resting state power.

Since the power distributions were skewed, we log-transformed the power values for statistical analyses.

### 2.6. Statistical Analyses

After exporting the ROI single trial time-series and pre-stimulus alpha to R, we restricted the time window of interest from −100 to 800 ms. Data from each ROI were analyzed separately, so we ran a total of 5 models. The models for the TPJs were different from those of the language-related ROIs.

In the TPJ models, continuous activation amplitude from the TPJ itself in the whole time window of interest was entered as the dependent variable. Main effects included the following:

- the condition (Metaphor vs. Literal) as factor;
- a non-linear effect of time, depending on the condition (this term captures the different changes in the activation over time in the two conditions);
- a non-linear effect of pre-stimulus alpha power from the TPJ itself, capturing the (possibly) non-linear modulation of activation amplitude by different magnitudes of pre-stimulus alpha;
- an interaction term, specifying the non-linear interaction of interest between the continuous variables of time and pre-stimulus alpha power, depending on the condition, was included to capture whether pre-stimulus power modulates, in a possibly non-linear way, the subsequent activations, in either of the two conditions. This term corresponds to the interaction of interest, which, in GAMMs, is modeled by a tensor smooth function, and allows us to answer the question regarding the influence of the pre-stimulus alpha power on the subsequent activation response within the TPJ.

In the models for the language-related ROIs, additional terms were included, since we wanted to test whether the continuous post-stimulus activation of each ROI (entered as the dependent variable) was better predicted by pre-stimulus alpha power recorded from the left or right TPJ, controlling for pre-stimulus alpha power from the same area. Therefore, in addition to the linear main effect of time, the non-linear main effect of time depending on condition, the non-linear main effect of pre-stimulus alpha power from the same ROI, and the interaction term specifying the non-linear interaction of interest between time and pre-stimulus alpha power depending on the condition within the same area (as in the TPJ models), we also added:

- a non-linear effect of pre-stimulus alpha power from the rTPJ;
- a non-linear effect of pre-stimulus alpha power from the lTPJ;
- an interaction term specifying the non-linear interaction between time and the rTPJ pre-stimulus alpha power, depending on the condition;
- an interaction term specifying the non-linear interaction between time and the lTPJ pre-stimulus alpha power, depending on the condition.

As a consequence, the language ROI models included three interactions of interest, while the TPJ models only included one. This allows us to compare the respective influence of the pre-stimulus alpha from the different locations (rTPJ, lTPJ, or the same ROI) on the post-stimulus activation. Results for the tensor representing the interaction involving the same-area pre-stimulus alpha are reported in the Additional Analysis in the OSF repository.

The R syntax for models with ROI activity on the TPJ as the dependent variable and for models with Language ROI activity as the dependent variable are reported in the Additional Analysis in the OSF repository.

In addition, we introduced a random structure for all the models, in order to reduce autocorrelation in the residuals and account for data dependency [93]. This included two random factor smooths: one for participants and one for each target word. To identify each trial uniquely, we created a factor variable called “Event” which comprised the combination of participant, condition, and target word. We used this variable as both a random intercept and slope, interacting with time. It is important to note that the target word was included in the random structure solely to model a portion of the random variability between trials and is not a variable of interest in the analysis. Therefore, it will not be discussed further. Additionally, we incorporated an AR1 model to account for autoregressive processes in the data. We estimated the value of rho by examining the model’s autocorrelation function (ACF) at lag 1 and then included it in our model along with a term specifying the starting point of each time series. The non-linear random structure of the models and the AR1 error model are essential for handling the intrinsic temporal dependency in time series data [93]. Since the model’s residuals were not normally distributed, we fitted the model with a link function for a scaled-t distribution [94]. To optimize computational time, we set the argument “discrete” to true, enabling more efficient processing. The models showed no convergence issues.

**Figure 3:**
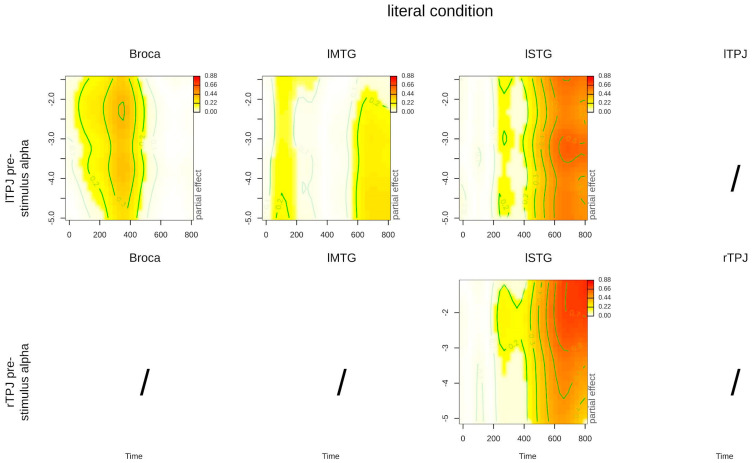
Contour plots of the main interaction between time and pre-stimulus alpha power for the literal condition, for significant effects. Time is represented on the x-axis, pre-stimulus alpha power level on the y-axis, and activation is color-coded. Colored blots represent plot regions where the interaction is significant, while white areas indicate regions where the confidence intervals (95% CI) around the predicted surface included zero, i.e., the interaction is not significant. Darker shades indicate greater activation.

**Figure 4:**
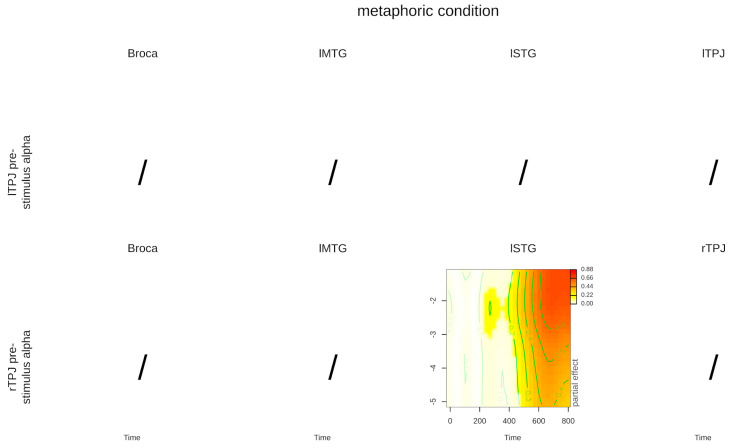
Contour plots of the main interaction between time and pre-stimulus alpha power for the metaphorical condition, for significant effects. Time is represented on the x-axis, pre-stimulus alpha power level on the y-axis, and activation is color-coded. Colored blots represent plot regions where the interaction is significant, while white areas indicate regions where the confidence intervals (95% CI) around the predicted surface included zero, i.e., the interaction is not significant. Darker shades indicate greater activation.

**Figure 5:**
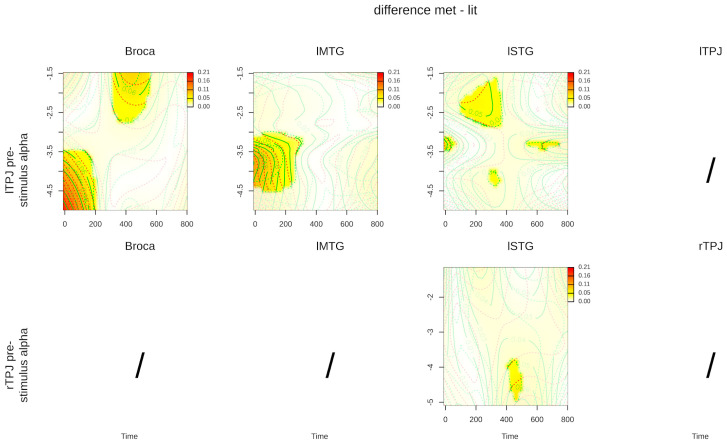
Contour plots showing activation differences between metaphorical and literal conditions for the main interactions of interest (time, alpha band power, and conditions). Time is represented on the x-axis, pre-stimulus alpha power level on the y-axis, and the difference in predicted activation is color-coded. Colored blots represent plot regions where the difference is significant, while white areas indicate regions where the confidence intervals (95% CI) around the predicted activation difference included zero, i.e., the difference is not significant. Darker shades indicate greater activation differences.

We inspected the significance of the interaction terms with the summary function, providing F statistics, degrees of freedom, and *p* values for each term (reported in Table 3 and Table 4). In GAMMs, the effects of the main interactions of interest are mainly interpreted through visual inspection of the tensor functions’ plots, which in our case are three-dimensional since they include two continuous predictors: time and pre-stimulus power (for more information on GAMM results visualization, please see [45]. As the sign of the results does not help to interpret the data (source activations have not expected signs as ERP components) we rectified the Z-transformed activation time series signal prior to plotting. Any departure from zero can thus be interpreted as “higher activation of the ROI”. Note that rectifying the signal is a common step in ROI pre-preprocessing (for more information please see the following link: https://neuroimage.usc.edu/brainstorm/Tutorials/SourceEstimation, accessed on 20 May 2023), but this was not performed in the GAMM analysis because rectification of the single-trial data led to distortions. The 3D plots are represented with time on the x-axis, pre-stimulus alpha power level on the y-axis, and color-coded activation that provides information on how the activation time series are modulated by pre-stimulus power depending on literal and metaphorical conditions. This interaction allows us to visualize the activation time course depending on pre-stimulus alpha levels and conditions, as shown in Figure 3 and Figure 4, but also to perform subtractions between the metaphorical and literal tensor surfaces as if they were activation time series. We followed this procedure because the time series difference allows us to isolate components of interest that are informative on the effects of the experimental manipulations [95]. Moreover, subtracting the metaphorical from the literal tensor surface also allows the identification of significant effects (predicted differences between activation time series) in relation to the pre-stimulus alpha level. These effects were calculated and plotted with the plot_diff2 function in the package itsadug [96] and are shown in Figure 5.

## 3. Results

For the language-related ROI models, results of the interactions between time, condition, and pre-stimulus alpha power recorded within these same areas can be found in the Additional Analysis in the OSF repository at the following link: https://osf.io/y2efz/?view_only=1be400a47cd44f32814d545978cfa09d.

The percentage of deviance explained by the models ranged between 10.3% and 15.4%.

Table 3 reports the significance of the interaction between time, pre-stimulus alpha power, and literal vs. metaphorical condition for the left and right TPJ. In these areas, the interaction was not significant. This means that, within the TPJs, pre-stimulus alpha power did not significantly predict the activation in any condition.

Table 4 reports the significance of the interaction between time, pre-stimulus alpha power, and the literal vs. metaphorical condition for the language-related ROIs. In the literal condition, lTPJ pre-stimulus alpha power predicted the post-stimulus activation in all the linguistic ROIs (Broca, lMTG, and lSTG), while rTPJ pre-stimulus power predicted only the post-stimulus activation in the lSTG. Instead, in the metaphorical condition, lTPJ pre-stimulus alpha power did not predict the post-stimulus activation in any of the language-related ROIs, while rTPJ pre-stimulus alpha power predicted only the post-stimulus activation in the lSTG. This means that pre-stimulus alpha power originating from the left and right TPJ significantly predicted the activation in both the literal and metaphorical conditions in different ways across areas (see Figure 2 and Figure 3).

As we can see from Figure 3, in the literal condition, lTPJ pre-stimulus alpha was associated with an early activation (about 200 to 400 ms) in Broca’s area, while in the lMTG and lSTG, it was associated also with activations in later time windows (after 400 ms). Within the lSTG, post-stimulus activations were also predicted by rTPJ pre-stimulus alpha in late time windows (after 400 ms), similar to the effects observed in association with lTPJ pre-stimulus alpha.

As shown in Figure 4, in the metaphorical condition, lTPJ pre-stimulus alpha power did not predict the activation in any language-related ROI, while rTPJ pre-stimulus alpha was associated only with late activations in the lSTG, in a time window resembling the effect in the literal condition.

Since time series differences allow us to isolate components of interest that are informative on the effects of the experimental manipulations [94] (see Section 2.6), we subtracted the predicted metaphorical activation from the literal one in each ROI, only for significant interactions. Figure 5 shows, for each ROI, the predicted activation difference between the metaphorical and the literal conditions depending on the left and right TPJ pre-stimulus alpha level.

lTPJ pre-stimulus alpha power predicted the activation differences between literal and metaphorical conditions in all the language-related ROIs, although in different time segments: lower levels of lTPJ pre-stimulus alpha power (depicted in the lower portion of the y axis of the figures’ panels) were associated with significant activation differences from 0 to 200 ms in Broca’s area and the lMTG. In Broca’s area, a higher pre-stimulus alpha (depicted in the higher portion of the y axis of the figures’ panels) was also associated with a significant activation difference around 400 ms. In the lSTG, differences were more attenuated and distributed, spanning almost the whole time window. Conversely, rTPJ pre-stimulus alpha predicted only a small activation difference around 400 ms in the lSTG.

To summarize, results show that left and right TPJ pre-stimulus alpha power did not predict post-stimulus activation within the TPJs themselves, but it predicted post-stimulus activations in the language-related ROIs in both the literal and metaphoric condition. More specifically, lSTG post-stimulus activation was predicted by both left and right TPJ pre-stimulus alpha power, while Broca and lMTG post-stimulus activations were predicted only by lTPJ pre-stimulus alpha. The contrast between conditions evoked a less strong pattern of results, with differences mainly in lTPJ response (Figure 5), possibly due to the role of this region in predicting post-stimulus activity in Broca’s area and the lMTG in literal but not in metaphorical conditions.

## 4. Discussion

**Figure 6:**
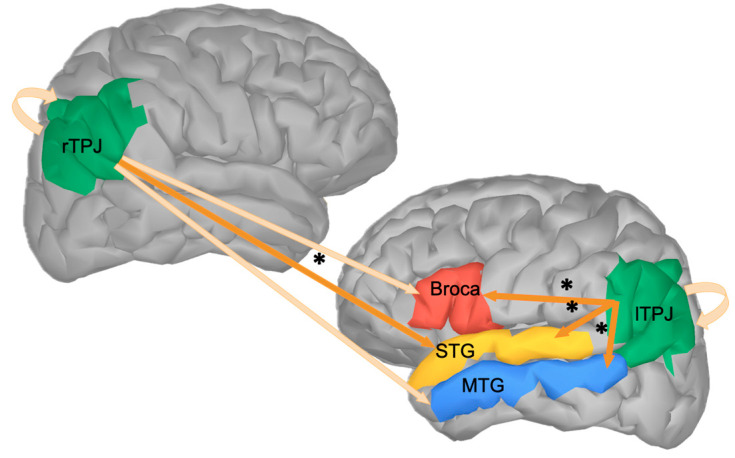
Summary of significant interactions of interest between pre-stimulus alpha power and post-stimulus ROI activations. Solid arrows marked with * represent significant effects, while lighter arrows represent the non-significant ones. Each effect is significant for different alpha values and different time points (see Figure 5 for details).

In the present study, we investigated whether the TPJs are involved in linguistic prediction, and we used Generalized Additive Mixed Models (GAMMs) to test whether spontaneous, trial-by-trial fluctuations of pre-stimulus alpha power recorded from the TPJs modulated the post-stimulus activations within the TPJs and in some core linguistic areas. We found that rTPJ predicted a difference around 400 ms in the lSTG. Conversely, lTPJ pre-stimulus alpha was associated with the post-stimulus activation of all the language-related ROIs under investigation (as summarized in Figure 6), and it also predicted early differences between metaphoric and literal conditions, especially in Broca’s area and the lMTG. In Broca’s area and in the lMTG, post-stimulus activation was also predicted by the pre-stimulus alpha power recorded within these same areas (see Additional Analysis in the OSF repository). Importantly, the analytic method used (i.e., GAMM) allowed us to observe that the TPJs had some influence in predicting post-stimulus activity of the linguistic areas, independently of the effects of the pre-stimulus power recorded in these areas (e.g., the left MTG was influenced by pre-stimulus alpha power in the lTPJ, but not by pre-stimulus alpha power in the MTG itself).

What is even more interesting is that within the TPJs, pre-stimulus alpha power did not predict the post-stimulus activation of the areas themselves: in other words, during the task, spontaneous fluctuations of pre-stimulus alpha power recorded in the TPJs appeared to modulate the activation in language-related ROIs, but not within the TPJs themselves.

These results indicate that, probably, the left and right TPJ have different roles in linguistic prediction, thanks to their functional connections with task-specific ROIs [2,29].

### 4.1. rTPJ

Our results showed a significant, albeit limited, effect of lower values of rTPJ pre-stimulus alpha power on lSTG post-stimulus activation visible around 400 msec (see end of Section 3 and Figure 5, lower panels). Evidence of the involvement of the rTPJ in literal [5] and figurative language processing has been previously reported [42,44] and is corroborated by the present findings. Generally speaking, one of the core functions of the rTPJ is attentional reorienting [12,13], but this region has been proposed to play a ubiquitous role in many cognitive domains [2]. Such a pervasive involvement of the rTPJ can be explained in terms of predictive processing and, in particular, through the concept of precision, i.e., the reliability of a prediction. As outlined in the Introduction, in a predictive processing view, attention can be seen as an emergent property of the precision optimization mechanism, which enhances expectations and prediction errors during the inferential process [65]. In other words, highly precise expectations could reduce the uncertainty associated with the underlying cause of upcoming stimuli (in our task, about the most plausible sentence meaning). In this way, cognitive resources are diverted towards more precise predictions [66], and the magnitude of expectation effects on neural activity depends on the precision of predictions and prediction error signals [67]. This mechanism has been traditionally interpreted as a higher allocation of attentional resources [66] and this “attentional” view is in line with evidence showing that cytoarchitectonic areas of the rTPJ, in particular, PGa, PFm, and PF, are sensitive to invalid predictions (invalidly cued trials) in an attentional task. These areas could be specifically recruited when subjects reorient towards internal or external salient stimuli or, in the present case, highly precise hypotheses [97]. We hypothesize that, in the time interval before the presentation of the final target word defining the sentence as literal or metaphoric, the rTPJ helps enhance predictions relative to the most plausible meaning of the sentence through a mechanism of precision optimization: highly precise expectations held by participants reduce the uncertainty associated with the most plausible sentence meaning and with lower levels of pre-stimulus alpha power; these precise predictions attract, in turn, cognitive/attentional resources [66], thereby enhancing the relative internal representations. When such precise expectations are tested against the actual sentence meaning in the post-stimulus interval, post-stimulus neural responses resulting from testing highly precise predictions against the actual stimuli are amplified proportionally to the level of precision of the predictions [67].

Our results suggest that post-stimulus neural responses in the lSTG are particularly sensitive to rTPJ pre-stimulus alpha levels, and especially so in the time window around 400 ms. This is consistent with previous EEG evidence showing that low levels of pre-stimulus alpha, associated with highly precise predictions, modulate the signal in the N400 time window following incongruent target words [45]. Combined with our results, this evidence supports the hypothesis that rTPJ alpha oscillations, probably associated with precision optimization mechanisms, influence how the upcoming stimuli are processed, and that within the language network, the lSTG might be particularly sensitive to precision optimization dynamics taking place in the pre-stimulus interval.

Our evidence for the involvement of the rTPJ in linguistic predictive processing is, however, limited, since we found that this area modulated post-stimulus activation only in the lSTG and for a very short time. The lTPJ, instead, exerted a more extended influence on all the linguistic ROIs under investigation.

### 4.2. lTPJ

Pre-stimulus alpha in lTPJ was associated with the post-stimulus activation of all the language-related ROIs under investigation (Figure 5, upper panels). The fact that pre-stimulus alpha power recorded from the lTPJ had more pervasive effects than pre-stimulus alpha power recorded from its right-hemisphere homologue, involving all the core language areas considered, is a relevant finding that can be interpreted along different lines. First, these more pervasive effects could be a consequence of signal propagation due to the contiguity of lTPJ, lMTG, and lSTG. However, the lSTG, the closest region to the lTPJ, reported the lowest activation in association with lTPJ pre-stimulus alpha, while activation in Broca’s area seemed to be stronger.

A predictive processing interpretation of the task may help us parsimoniously explain the overall pattern of results. In our task, to correctly make sense of the sentence stimuli (in the case of metaphor stimuli, to infer the figurative meaning beyond the literal one), participants generated hypotheses based both on local sentence information and their previous knowledge activated by the sentence content [38,39,40]. Although their prior knowledge guided linguistic processing, participants may have held some degree of uncertainty about the correct meaning of the sentence [31] until they tested the hypothesis on the upcoming stimulus against the actual input, i.e., the last target word that disambiguated the meaning of the sentence. In the context of these considerations, our results could indicate that in the time interval preceding the target word, the lTPJ is involved in the process related to the generation of internal models of the sentence based on prior world knowledge, which has a major role in guiding interpretations in conditions of uncertainty (see Section 1.2.4). Internal models’ predictions are then tested against target words, generating neural responses in task-specific areas, namely, Broca, the lMTG, and the lSTG. The pre-stimulus alpha effect could reflect spontaneous fluctuations of prior precision of the hypotheses on upcoming stimuli.

Interestingly, the broad effects of the lTPJ varied across areas. In particular, lTPJ pre-stimulus alpha predicted early differences between metaphoric and literal conditions in Broca’s area and the lMTG. Moreover, in Broca’s area, lTPJ pre-stimulus alpha was associated with significant differences around 400 ms, while in the lSTG, effects were significant also in a later time window spanning from 500 up until almost the end of the time window (see Figure 5, upper panels). This sequence of effects is consistent with previous EEG evidence from other metaphor and figurative language comprehension studies. For example, various studies on metaphor comprehension found a significant effect as early as ~200 ms after the processing of the target word [98,99]. Effects in this time window have been associated also with irony comprehension, another form of figurative language [100,101], and with sentence constraint and predictability of the target word [102,103,104], suggesting that internal models’ predictions influence the processing of metaphorical targets starting from very early stages after presentation. We can therefore hypothesize that the early activations in all the language areas under investigation might reflect early processing of metaphorical targets.

Furthermore, the effects around 400 msec and later, found in Broca’s area and in the lSTG respectively, are in line with other EEG studies of metaphor comprehension that have detected N400/late positivity complexes [34,83,105]. The activations around 400 ms found in Broca’s area probably reflect the prediction errors that follow the mismatches between predictions and metaphorical target words, similar to the N400 ERP found in EEG studies and to its MEG analogous, the N400m [106,107,108,109]; in turn, the later activation spanning from 500 ms to almost the end of the time window found in the lSTG could have its counterpart in the P600 ERP [108,110,111] and might represent the later attempts to make sense of the sentence through processes like reanalysis, repair, or reinterpretation, in line with the late positivities observed in violation paradigms [32].

Taken together, the timing of modulations of post-stimulus responses by lTPJ pre-stimulus alpha in the three language ROIs suggests that the language areas under investigation could be particularly sensitive to the early phases of prediction testing that occur when the target stimulus is read, and in particular during the processing of metaphorical targets and later attempts to make sense of unexpected (metaphorical) inputs.

It is important to note that the analysis on separate conditions highlighted a more extensive pattern of effects from the lTPJ in the case of literal than in the case of metaphorical sentences. While this finding requires further investigation, we might speculate that, while literal sentences engage a broad range of linguistic prediction mechanisms, metaphorical sentences—whose results are robust for the rTPJ—possibly rely on the integration between general world knowledge and local sentence information [17,18], as well as on a broader set of functions than language, including mind reading and executive and associative aspects, in addition to multimodal components such as imagery [42,112]. This speculation is substantiated by accumulating evidence about the role of the TPJs as multimodal integrative and domain-general predictive hubs (reviewed in the Introduction, Section 1.2), as proposed by the nexus model [21], stating that the TPJs integrate information from various cognitive domains to support decision-making, or by the contextual updating hypothesis, proposing that TPJs have the function of updating internal models of the environment [22]. More recently, in the same vein, the TPJs have been hypothesized to take part in a domain-general prediction network thanks to their structural and functional connections to domain-specific areas [1,29], so that the pathological disconnection of the TPJs from specific networks could give rise to cognitive and neurological symptoms [2].

### 4.3. TPJs and Different Aspects of Predictive Processing

To sum up, our results indicate that the left and right TPJ exerted different influences on the post-stimulus activation of the various language-related ROIs. As we outlined in Section 4.1 and Section 4.2, these differences may depend on the respective sensitivity to different aspects of predictive processing. On the one hand, the rTPJ, traditionally linked to attention, might be more sensitive to precision dynamics that enable the allocation of attentional resources to more precise predictions. These precision-related dynamics, reflecting on pre-stimulus alpha power, modulated only the activation in the lSTG in a short time window around 400 ms, probably reflecting the enhancement of linguistic prediction error as an effect of highly precise expectations [45,65,66,67]. On the other hand, the lTPJ seems to have a role that precedes the prediction testing phase, i.e., in the construction and updating of an internal model of the sentence that has to be tested against the final target word [17,18], a process that extensively involves all the language-related ROIs under investigation, and causes modulations more extended in time. This might reflect a more holistic interpretation of the sentence stimuli after the presentation of the target words, starting from early processing to the generation of prediction errors and their relative reanalysis and possibly repair [32], allowing for the correct interpretation of the sentence, particularly of metaphors.

In sum, our results showed that both right and left TPJs are involved in linguistic prediction, during both literal and figurative sentence comprehension, thanks to their functional connections with language-related areas. Importantly, we showed that the activity of the TPJs seemed to precede the activity in other areas, supporting the idea that its role is actually related to predictive processes. We suggest that both TPJs could function as predictive hubs, bringing together various cognitive processes and types of information while closely interacting with other brain regions and networks [1,2,29]. Following this line of reasoning, functional connections between domain-general and task-specific regions are crucial for predictive computations in various cognitive domains, and the specific functions of the TPJs may be determined by the networks activated during ongoing activities, such as the language network in this case. As a result, the context- and network-dependent nature of the TPJs facilitates the seamless integration between different brain regions and cognitive processes, enabling context-specific networks to effectively utilize their integrative and contextual-updating capabilities in various behavioral and cognitive scenarios.

Limitations. There are some limitations in the present study. First, we did not directly manipulate the level of sentence constraint, with the consequence that the level of predictions’ precision fluctuates spontaneously across stimuli, not allowing us to systematically link different levels of alpha power to different levels of constraint and precision. Explicitly addressing the manipulation of sentential constraint could therefore lead to a clearer picture of how more or less precise predictions affect the subsequent stimulus processing. In relation to this, we point out that in language experiments (and the present one makes no exception), it is often difficult to rule out the role of uncontrolled properties of the stimuli in the observed effects. More specifically, our pre-stimulus interval also included the indefinite article, and there is evidence that anticipatory processes might be happening already at this level [91], which might influence the level of pre-stimulus alpha power and, in turn, the final results. To explore this issue, we ran control analyses based on simulations, which can be found in the Additional Analysis in the OSF repository. These additional analyses demonstrated that no experimental item was associated with a particular value of alpha, and therefore the effects we report were not due to uncontrolled properties of the stimuli, but to random fluctuations. It is also important to note that although we showed a relationship between the activity in both TPJs and language-related areas, the nature of MEG data (as a neurophysiology technique) is purely correlational. Future studies perturbing TPJ activity in tasks and co-registering activity in other areas (e.g., TMS-EEG studies) could further corroborate the interpretations of the results put forward in the present study. In addition, acknowledging that prediction is a continuous and ongoing process, it is plausible that the TPJs exert a continuous influence on other brain areas throughout the sentence, and that the TPJs’ alpha level could influence the subsequent activation in the language-related areas during a time interval spanning the whole sentence. In the present study, we took into consideration only specific time intervals at the end of the sentence. Future studies could adopt a more complex “sliding window” approach to consider the influence of pre-stimulus alpha on subsequent activation, which would further clarify the complex dynamics of linguistic prediction.

A final consideration concerns the interpretation of the roles of the left and right TPJ. In this article, we showed how pre-stimulus alpha power in these areas may be associated with spontaneous fluctuations in prior precision. However, the results do not indicate that this is the only function of the TPJs. These areas have also been implicated in other aspects of predictive processing, such as contextual updating [22,23,24] and hypothesis generation [29], potentially involving different neurophysiological mechanisms not explored in the present study (e.g., beta-band activity for hypothesis generation).

## 5. Conclusions

The goal of the present study was to answer the following questions: are both TPJs involved in linguistic prediction? Can spontaneous fluctuations of pre-stimulus alpha (associated with prior precision) recorded in the TPJs modulate the subsequent brain responses to target stimuli? If so, is this modulation local, i.e., limited to the TPJs, or can pre-stimulus TPJ activity influence post-stimulus activations in other task-related, i.e., linguistic, areas? Finally, do the left and right TPJ exert different kinds of influence on post-stimulus responses recorded from linguistic ROIs?

Through MEG recordings and an innovative statistical method (GAMMs), we showed that spontaneous fluctuations of pre-stimulus alpha power in both TPJs were associated with post-stimulus activity in language areas. This possibly indicates that the TPJs are involved in prediction generation during a sentence comprehension task, both literal and metaphorical, thanks to their functional connections with language-specific areas. The left and right TPJ are probably involved in linguistic predictive computations with different roles, as they exert a different degree of influence on the post-stimulus activities of language-related brain regions, depending both on the type of stimulus and on the area under investigation.

These findings represent the starting point for bridging the gap in the existing literature about temporal and spatial dynamics of linguistic predictive processing, by highlighting the interplay and possible functional connections between key regions of the language network and the bilateral TPJs.

## Figures and Tables

**Table 1:** Examples of stimuli, translated from original Italian.

Condition	Stimulus	Related Adjective	Unrelated Adjective
Literal	That weapon is a bomb	Explosive	Difficult
Literal	That animal is a bear	Unsociable	Fitting
Literal	That tree is an oak	Strong	Nautical
Metaphor	That show is a bomb	Explosive	Difficult
Metaphor	That sailor is a bear	Unsociable	Fitting
Metaphor	That marriage is an oak	Strong	Nautical
Filler	That city is a metropolis	Expanded	Credible
Filler	That cup is a trophy	Prestigious	Friendly
Filler	That appliance is a blender	Homely	Inebriating

**Table 2:** MNI centroid coordinates of the selected ROIs.

ROI	x	y	z
Broca	−51	9	12
lMTG	−59	−34	−13
lSTG	−56	−10	−11
lTPJ	−45	−70	37
rTPJ	45	57	39

**Table 3:** *F* statistics, effective degrees of freedom (edf), and *p* values of interactions between time, power, and condition for the TPJs. There were no significant results.

	lTPJ		rTPJ	
	*F* (edf)	*p* Value	*F* (edf)	*p* Value
Interaction: time, power, literal condition	0.186 (1.244)	0.654	1.879 (2.392)	0.119
Interaction: time, power, metaphor condition	3.046 (1.007)	0.080	0.754 (1.005)	0.385

**Table 4:** *F* statistics, effective degrees of freedom (edf) and *p* values of interactions between time, power, and condition for the language-related ROIs. Significant results are marked with *.

	Broca		lMTG		lSTG	
	*F* (edf)	*p* Value	*F* (edf)	*p* Value	*F* (edf)	*p* Value
Interaction: time, lTPJ power, literal condition	4.683 (1.008)	0.031 *	6.150 (1.023)	0.014 *	2.811 (5.297)	0.006 *
Interaction: time, lTPJ power, metaphor condition	2.156 (2.984)	0.069	0.837 (9.84)	0.632	1.113 (1.008)	0.289
Interaction: time, rTPJ power, literal condition	0.572 (3.295)	0.729	0.658 (2.523)	0.607	3.397 (2.735)	0.021 *
Interaction: time, rTPJ power, metaphor condition	1.319 (8.879)	0.195	1.947 (5.859)	0.059	3.441 (1.836)	0.030

## Data Availability

Data can be shared upon reasonable request to the Corresponding Author, and in accordance with Italian regulations for privacy of biomedical data.
